# Understanding the Impact of Subsidizing Artemisinin-Based Combination Therapies (ACTs) in the Retail Sector – Results from Focus Group Discussions in Rural Kenya

**DOI:** 10.1371/journal.pone.0054371

**Published:** 2013-01-14

**Authors:** Sarah V. Kedenge, Beth P. Kangwana, Evelyn W. Waweru, Andrew J. Nyandigisi, Jayesh Pandit, Simon J. Brooker, Robert W. Snow, Catherine A. Goodman

**Affiliations:** 1 Malaria Public Health Group, Kenya Medical Research Institute -Wellcome Trust Research Programme, Nairobi, Kenya; 2 Division of Malaria Control, Ministry of Public Health and Sanitation, Nairobi, Kenya; 3 Pharmacy and Poisons Board, Nairobi, Kenya; 4 London School of Hygiene and Tropical Medicine, London, United Kingdom; 5 Centre for Tropical Medicine, Nuffield Department of Clinical Medicine, University of Oxford, Oxford, United Kingdom; Tulane University School of Public Health and Tropical Medicine, United States of America

## Abstract

**Background:**

There is considerable interest in the potential of private sector subsidies to increase availability and affordability of artemisinin-based combination therapies (ACTs) for malaria treatment. A cluster randomized trial of such subsidies was conducted in 3 districts in Kenya, comprising provision of subsidized packs of paediatric ACT to retail outlets, training of retail staff, and community awareness activities. The results demonstrated a substantial increase in ACT availability and coverage, though patient counselling and adherence were suboptimal. We conducted a qualitative study in order to understand why these successes and limitations occurred.

**Methodology/Principal Findings:**

Eighteen focus group discussions were conducted, 9 with retailers and 9 with caregivers, to document experiences with the intervention. Respondents were positive about intervention components, praising the focused retailer training, affordable pricing, strong promotional activities, dispensing job aids, and consumer friendly packaging, which are likely to have contributed to the positive access and coverage outcomes observed. However, many retailers still did not stock ACT, due to insufficient supplies, lack of capital and staff turnover. Advice to caregivers was poor due to insufficient time, and poor recall of instructions. Adherence by caregivers to dosing guidelines was sub-optimal, because of a wish to save tablets for other episodes, doses being required at night, stopping treatment when the child felt better, and the number and bitter taste of the tablets. Caregivers used a number of strategies to obtain paediatric ACT for older age groups.

**Conclusions/Significance:**

This study has highlighted that important components of a successful ACT subsidy intervention are regular retailer training, affordable pricing, a reliable supply chain and community mobilization emphasizing patient adherence and when to seek further care.

## Introduction

Malaria remains a critical health problem, with over 90% of malaria deaths reported in Africa [1]. Artemisinin-based combination treatments (ACTs) are generally accepted as the best treatment for uncomplicated malaria, and have been adopted throughout Africa in national treatment policies, but their use remains low [1], [2], [3]. Reasons for this include their high price, making the drugs inaccessible to those of low socio-economic status [4]; and frequent stock-outs in public sector facilities [5], [6], [7], [8]. In addition, quality of care is compromised by the poor counselling advice given by ACT providers on how the drugs should be utilised [8], [9]; low use of diagnostics to ensure targeted treatment of malaria [1], [3], [10]; utilisation of artemisinin monotherapies which may encourage development of artemisinin resistance [7], [11]; and poor adherence to dosing guidelines by both health workers [10] and consumers [12], [13], [14].

There is considerable interest in the potential of private sector antimalarial subsidies to increase coverage of effective malaria treatment and expand access to ACT [15], [16], [17], with the Affordable Medicines Facility – malaria (AMFm), an ACT subsidy managed by the Global Fund to fight AIDS, TB and Malaria, currently being implemented in eight malaria endemic countries [18]. The key aims of such subsidy programmes are to increase ACT availability, affordability and use, “crowding out” non-recommended treatments from the market.

Prior to the roll out of AMFm, a cluster randomized trial of such ACT subsidies was carried out in a high malaria transmission area of rural western Kenya. The pilot subsidy programme involved the provision of subsidized artemether–lumefantrine (AL) for children under five years through the private retail sector, branded as Tibamal, combined with retailer training and community awareness activities. Quantitative results from household, provider and mystery shopper surveys demonstrated a substantial increase in access to and coverage of ACTs [19], [20], [21]. The intervention led to an increase in the proportion of outlets stocking AL in the intervention arm from 2.4% at baseline to 37.6% at follow up [19]. There was also a marked decrease in the average time taken by caregivers in the intervention arm to access shops that dispensed AL from 32 minutes at baseline to 10 minutes at follow up [22]. This was associated with a significant increase in the proportion of children aged 3–59 months receiving prompt treatment with AL, from 14.1% to 35.9% [21]. Furthermore, both the household and mystery shopper surveys showed that over 95% of retailers adhered to the recommended retail price for subsidised ACT [20], [21]. Diversion of the subsidised drug to non-target age groups was reported to be low, with Tibamal being used for only 6.7% of fevers in those 5 years and above in the intervention arm [23].

However, the quantitative results also highlighted limitations that may have decreased the intervention's impact. Coverage of AL was still well below the Roll-Back Malaria target of 80% of fevers being treated with prompt effective treatment [2], [21]. In addition, only 37% of retailers stocked AL at follow-up; although this was much higher among Tibamal trained outlets (72%), it was still far from universal [19]. Mystery shopper surveys showed that only 25% of intervention arm outlets dispensed AL at follow up, and they were poor at giving appropriate advice to clients. Less than 35% of outlets dispensing AL gave appropriate advice on how to administer the drugs or on what to do if the child did not feel better. Advice was particularly poor on appropriate foods to give with the drugs (12%), and on what to do if the child vomited (3%) [20]. Although shopkeepers were supposed to refer children with severe disease or adverse drug reactions (ADRs) to a health facility, almost no reports of referrals were recorded in routine records [21]. Furthermore, adherence to AL was sub-optimal, with only 67% of children consuming the correct dose at follow up in the intervention arm, although adherence to Tibamal was not any worse than adherence to AL obtained through health facilities [21]. Finally, as might be expected given that diagnostics were not part of the intervention package, very few caregivers (8%) reported that their febrile children received a malaria diagnostic test [23].

It is important to understand why these successes and limitations occurred in order to learn lessons for AMFm and other similar subsidy programs involving the retail sector. To address this we conducted focus group discussions (FGDs) with retailers and caregivers who had participated in the Tibamal pilot in order to gain insights into the quantitative findings. This is the first study, as far as we are aware, to present qualitative data on experience with retail level ACT subsidies.

## Methods

### Study Sites

The study was conducted in three districts in Western province: Busia, Butere-Mumias and Teso. This area was selected because of its high malaria endemicity; the presence of a relatively active retail market; and the absence of other malaria treatment interventions.

The percentage of the population living below the poverty line was 67% in Busia, 62% in Butere-Mumias and 50% in Teso, with population densities per km^2^ of 433, 611 and 406 respectively [24]. The percentage of household heads who had completed primary school was 57% in Busia, 54% in Teso and 58% in Butere-Mumias [24]. Western Kenya suffers from the highest malaria prevalence in Kenya, with *P. falciparum* parasitaemia prevalence in children aged 2 to 10 years greater than or equal to 40% [25]. At the time of the survey, Butere-Mumias had 51 health facilities, Busia 39 and Teso 21, consisting of dispensaries, health centres and one district hospital per district [26]. All government health facilities in Kenya were supposed to supply AL for free to patients, although stock-outs and unofficial fees were common [5], [27]. Malaria diagnosis was predominantly clinical in both public and private health sectors [9], [28].

### Intervention Design

The study employed a pre-post randomized cluster controlled design, and the intervention was implemented at the sub-location level (a sub-location is the fifth and lowest administrative level in Kenya). Three control and three intervention sub-locations were randomly selected within rural areas of each district, with a buffer zone of at least two sub-locations between intervention and control sub-locations to minimize contamination. No interventions were implemented in the control sub-locations. In both intervention and control areas the policy of provision of free AL at government facilities continued unchanged.

The intervention package was designed and implemented by the Division of Malaria Control (DOMC) in collaboration with Population Services International (PSI), the Pharmacy and Poisons Board (PPB), and Ministry of Health staff at the province and district level. Following extensive formative research and pre-testing, PSI and the DOMC developed a branded pre-packaged AL product for the treatment of malaria in children in two pack sizes: a yellow six tablet pack for 3 months to less than 3 year olds (5–15 kg) and a blue 12 tablet pack for 3 to 4 year olds (15–25 kg). Additional consumer friendly information was added to the product's outer packaging using pictorials and written instructions to promote appropriate dosing, in a form suitable for those with low literacy levels. The product's instructions also included details on the Integrated Management of Childhood Illnesses (IMCI) danger signs and the need to refer to public health facilities severe conditions and children under three months. The AL was branded as Tibamal, a pretested name derived from the Kiswahili words ‘Tiba ya Malaria’, meaning *malaria cure*. AL was registered in Kenya as a prescription only medicine, however special dispensation was granted by the PPB for AL to be sold as an over the counter medication in the intervention areas.

PSI sales staff delivered the treatment directly to selected retail outlets on a monthly basis at a subsidized wholesale cost of 0.10 USD (Ksh. 8) per treatment pack, both packs being the same price (Source of exchange rate: http://www.exchangerate.com/past_rates_entry.html. Accessed 13/4/2010. On 1st November 2008, when the subsidized drugs were first distributed, 1 US dollar was equivalent to 81.23 KSH). The outlets were instructed to sell the packs at a retail price of 0.25 USD (Ksh. 20), and this price was printed on the drug packaging. The retail price was designed to be competitive with less effective monotherapies such as SP and amodiaquine, which were sold at around 0.37 USD (Ksh. 30) per adult dose. The average retail price of AL without the subsidy was around 6.16 USD (Ksh. 500) per adult dose.

**Figure 1:**
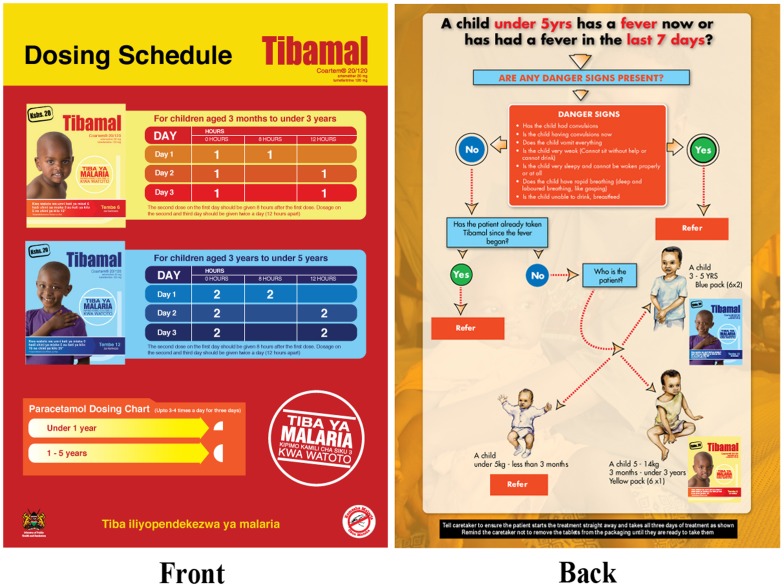
Job aid provided to retailers. In intervention outlets, retailers were provided with a treatment job aid. The front of the job aid provided a dosing guideline for each of the Tibamal treatment packs and the corresponding Paracetamol dosing. The back of the job aid was a flow chart on potential danger signs to ask the caregiver about to determine if there was need for referral or the treatment to provide.

Outlets had been identified from a baseline retail census and selected for inclusion into the intervention if they sold either an antimalarial or antipyretic in the past year. A total of 225 outlets were selected in the intervention areas, of which 61 were specialized drug stores (registered or unregistered pharmacies) and 164 general stores which sold medicine alongside general household goods. The majority of drug stores staff had a clinically-related qualification (73%), but this was very rare among general store staff (2.7%) [22]. Outlet staff attended a one day malaria-related training offered between August to October 2008 covering clinical diagnosis, treatment, ADRs and patient referral. Training materials were developed by the implementation team, building on those used previously for retailer training in Kenya [29]. In addition, retailers were supplied with job aids including simple treatment algorithms for AL and paracetamol to improve the quality and quantity of information provided to consumers (Figure 1). Retailers were also supplied with a Daily Activity Register (DAR) book in which they were to record the name of the child for whom the drug was bought, their age, their homestead head's name and the pack of Tibamal dispensed. Subsidized AL was supplied to outlets from November 2008. Supportive supervision of the retailers by PSI took place in February 2009, to assess if outlets were practicing as trained.

A series of promotional activities in the intervention areas and related dominant market centres began in March 2009. Messages targeted caregivers of children under five and promoted the benefits of AL and its availability both in public sector facilities and identified private sector outlets. Messages were delivered through small group sessions and community leader workshops, reinforced by point of sale materials, posters, leaflets and localized media such as wall paintings. Branded caps, t-shirts, headscarves, pens and calendars were distributed. Above the line communication strategies (newspapers, television and radio) were not used in this pilot to avoid possible contamination between intervention and control areas. In the event of a treatment failure or ADR retailers were instructed to refer caregivers to the nearest health facility using Community Health Worker (CHW) referral forms.

Quantitative evaluation of the intervention took place through household, provider and mystery shopper surveys conducted at baseline (June-August 2008) and endline (June-August 2009) in the 18 sub-locations [19], [20], [21].

### Qualitative Data Collection and Analysis

Eighteen FGDs were conducted in February-March 2010 in all the 9 intervention sub-locations. The FGDs therefore took place 16 months after the start of drug distribution, 11 months after the start of community awareness activities and 6–8 months after follow-up quantitative data collection, though the intervention was still being fully implemented at the time of qualitative data collection. In each sub-location a group of shopkeepers and a group of caregivers of children under five were interviewed separately. To ensure that a range of experiences were presented, shopkeepers were purposively selected to include a balance between general and specialized drug stores, and variation in behaviours recorded during the provider and mystery shopper follow up surveys. These behaviours included whether someone from the outlet had attended Tibamal training, whether they were stocking Tibamal, how well they gave advice, and the price at which they dispensed Tibamal. Caregivers were purposively selected based on treatment seeking behaviours recorded in the follow-up household survey, such as whether they had used Tibamal, their education and socio-economic level, and their choice of treatment provider. Invitation letters were sent through the local administration to retailers and to the household heads of caregivers. Consent to participate in the Focus Group Discussions and for digital recording was obtained verbally from the participants after an information sheet was read in vernacular language and paraphrased by the moderator in Swahili. One participant was then nominated by the group to sign the consent form on behalf of the whole group as a written confirmation of consent before the discussions began.

Each group had between 6–10 participants. The retailers were evenly balanced between females (54%) and males, and the majority were from general stores (65%), reflecting their predominance in the quantitative surveys. Amongst caregivers, all were female, with over 95% being mothers of children below 5 years, and the remainder being grandmothers. The mean age was 36 and 31 years amongst retailers and caregivers respectively. The majority of retailers (55%) had some secondary school education whereas the most caregivers (77%) had primary school education only.

Discussions were based on pre-prepared interview guides, which were piloted prior to fieldwork. All FGDs were carried out in Swahili though participants were encouraged to answer in the language they felt most comfortable with (English, Swahili and/or local languages). The interviews were conducted by research staff, accompanied by two trained fieldworkers per district with previous qualitative data collection experience and local language skills, who participated as note-takers. The interviews were recorded using digital tape recorders.

The guides were designed to capture information on retailers' and caregivers' experiences of the intervention. Retailers were asked about the benefits and costs of being involved in the programme, and any problems experienced in the implementation process. Caregivers were asked about their opinions on treatment sources, availability of products, barriers faced in seeking treatment, and any concerns they had regarding the programme. The (mis)use of intervention AL in adults and older children was also explored. Further information was obtained on the use and sale of diagnostics, and participants were shown malaria rapid diagnostic tests (RDTs) and provided with an explanation of their use.

The interviews were translated and transcribed in English, and the accuracy of the transcripts checked against the audio files. Field notes and observational data were incorporated into the transcripts but handled separately during analysis. Transcripts were imported into NVivo 8 software (QSR International Pty Ltd, 2008) for coding. An initial coding tree was developed using themes from the interview guides and previous literature. Two researchers separately coded about 20% of the transcripts and discussed and agreed on the final coding tree, incorporating new emerging codes. The final coding tree was used as an organizational framework in which data were summarized into each key node and salient features highlighted. Within nodes responses were compared between retailers and caregivers, and any links between nodes were identified.

### Ethical Approval

Ethical approval was obtained from the Kenya Medical Research Institute Ethical Review Committee (# 1361), the Kenya Pharmacy and Poisons Board Ethical Committee for Clinical Trials (# PPB/ECCT/08/07), and the London School of Hygiene and Tropical Medicine Ethical Review Committee (# 5288). The study is registered with the International Standards Randomized Controlled Trial Number (# ISRCTN59275137).

## Results

The results begin with presentation of the perceptions of retailers and caregivers on the components of the intervention, and how these were linked to the positive outcomes observed. We then draw on the FGD data to identify potential factors that may have limited the success of the intervention.

### Perceptions of intervention components

We investigated perceptions of the five key components of the intervention: shopkeeper training, promotional activities, provision of job aids, drug packaging and drug pricing.

#### Shopkeeper training

As described above, the selected shopkeepers attended a one day malaria-related training covering clinical diagnosis, treatment, ADRs and patient referral. A large majority of retailers said the training was beneficial. They specifically mentioned gaining knowledge on dispensing drugs, advising clients, and recognizing the symptoms that required treatment and referral, increasing their confidence in providing treatment.

*“It was good because when dealing with drugs you can't just come from anywhere and start giving them because you will mess up. You can give an overdose or under-dose; to me I saw it was good that they first went to educate people, so they understand before giving them drugs. That was okay.” Retailer, Teso.*

Although they cited numerous benefits from the training, most retailers noted that the one-day training was *“too short”*, many stated that the financial allowance provided (Ksh. 500 = 6.16 USD) was too low to compensate for missing a day's business, and several complained that not all attendees were genuine drug sellers.

#### Promotional activities

The majority of caregivers and retailers reported satisfaction with the promotional activities, saying that they increased awareness of the drug, leading to a marked increase in volumes sold. It was further agreed by almost all retailers and caregivers that as a result all community members, young and old, down to the “*grassroots”* had heard about Tibamal. A few retailers added that *“rigidity”* and strong beliefs regarding other antimalarials were decreased with increasing knowledge of Tibamal.

*“These promotions have accelerated awareness…that is why you see the demand is very high, because a good number of people have these T-shirts and as they walk around they are creating awareness….people asking “what is this?”….and also the shops that are selling, even if they are 10 in a place, at least 2 shops were being painted with Tibamal colours, which …also accelerated consumption.” Retailer, Busia.*

Almost all caregivers reported that through the promotional activities they had learnt the symptoms that warrant Tibamal use which made them more confident. They also learnt where to buy Tibamal and the importance of taking prompt action when their children got sick. Promotional items such as the headscarves, calendars and posters, helped them learn the difference between the packs and reminded them how to use the drugs.

*“It helps us too in our knowledge….to understand issues quickly… if you are given a t-shirt or a handkerchief you will see the word Tibamal and what does it mean? Tibamal means a drug. If it is a calendar, you will look at it and know… if I am given drugs at this time or on this day, I am supposed to use the drugs as is expected… I look at the date, and see how I can give the drugs to my child.” Caregiver, Busia.*

A few caregivers said that the promotional activities clarified the correct pricing for Tibamal. They noted that when Tibamal first appeared on the market a few retailers tried to sell the drugs above the recommended price, but the promotions had made community members aware of this. Retailers concurred, stating that role plays during promotional activities had warned them that anyone selling above the recommended price would be banned from selling the drugs.

A further benefit mentioned by many retailers was that the promotions informed caregivers which retailers had been trained and stocked Tibamal, and that the outlet painting/branding made the shops easily identifiable and *“smart”*.

*“…because of the painting of the shops…people just need to look and see “Oh! Tibamal” they will then ask there first.” Retailer, Butere-Mumias.*

The only negative view frequently expressed on the promotion was a feeling by retailers that they were not sufficiently consulted and had been *“sidelined”* and left out of the promotional activities.

*“….mostly these retailers were not that much involved in those plays and what not. But they should have been on the front line because they are the ones mostly selling these drugs to people there.” Retailer, Teso.*

The promotional activities and shopkeeper training appeared to have led to high levels of knowledge about Tibamal, and positive perceptions of its effectiveness. Tibamal was very well known amongst participants, and the majority of retailers and caregivers knew the indication for Tibamal was fever and/or malaria. Most retailers stated that the community members knew that Tibamal was the “*right drug”*.

*“…it is near to us, and when you use it, you get treatment and that is even why it is called Tibamal, because it treats; so I am very thankful.”Caregiver, Teso.*

*“….the goodness of Tibamal….malaria drugs are many, but….Tibamal works very fast. For example, if a child is in bad shape if you give the child the medicine and she takes it well, after 15 minutes the child will be okay….” Retailer, Teso.*

Very few retailers and caregivers reported having seen or heard about children experiencing side effects when taking Tibamal. It was unanimous that Tibamal was very popular and that the drugs sold *“like fried groundnuts”*, meaning at a very high rate. Retailers highlighted that the drugs sold even faster when government health facilities experienced stock outs of AL. Caregivers felt they could trust the quality of Tibamal as it was *“fast moving”* unlike other drugs that could stay in stock for over a year and hence not work effectively. The retailers stocking Tibamal added that the sales were very profitable, and had a positive impact on sales of other items.

*“…. you get that someone leaves home with the intention of buying the drug but when they reach the shop, you find that they think “eh! my child may fear taking the drug, so let me get them some juice or mandazi (a fried snack)”…. so you find that other shop has mandazi and yours has mandazi but in your shop they will get finished quickly.” Retailer, Teso.*

#### Job aids

Most retailers said during FGDs that they used the job aid with dispensing instructions, though this was surprising as provider survey data showed that only 44% of trained outlets had one [19]. They mentioned that it served as a reminder and helped them administer drugs and identify those in need of referral, being particularly helpful to retailers without a medical background. They added that it helped when explaining to their customers how to administer the drugs, especially clients with limited or no education, as they could use the pictures to direct them. A few retailers said that when they left someone in the outlet who had not attended the training, they could refer to the job aid if they had to dispense Tibamal.

*“…it is the key to the use of that drug.” Retailer, Teso.*

No retailers reported major difficulties when using the job aid, though many stated that it was not easy for those who had difficulty reading. A few added that having the chart in English only posed a challenge and suggested that it be available in Swahili and local dialects, and also enlarged with the information printed in a larger font. Many requested that the information on paracetamol should include syrups as well as tablets.

Experience with the daily activity register (DAR) was more mixed. All retailers selling Tibamal reported using it, most viewing it as a means for both PSI and the retailers themselves to monitor their stock and Tibamal sales, as a basis for being resupplied. They added that having *“a government stamp and logo at the top”* of the forms legitimized their work and made the community trust them.

However, most retailers reported difficulties with recording all the required details in the DAR because they were busy and because some customers were reluctant to provide the information, or did not know the name of the sick child. This led to problems in balancing their stock records such that some confessed to *“just filling in”* (i.e. making up) the entries if the missing records were few.

*“we forget, you could be asking about a child, then another customer comes; you find yourself saying ”I will fill it in later” then you don't get a chance to do it.” Retailer, Teso.*

These difficulties were compounded when they left someone to stand in for them in their outlets who was illiterate. However, most retailers said they ensured their DAR records were in order because as one stated *“if you make a mistake filling those forms of theirs, you will not sell again”*. The same retailer confirmed that she and her husband had been stopped from selling the drugs as there was an instance when they had failed to fill in about ten entries.

#### Tibamal packaging instructions

Consumer friendly information was included on the Tibamal packaging with pictures and instructions on how the drugs should be taken. The majority of caregivers said the instructions were easy to understand and particularly helpful on how to grind up the drugs for very young children who had difficulty swallowing tablets, and what foods to give with the drugs. They felt that the information was more helpful than that provided by health facility staff, where it was often *“just written 1×2”*, combined with verbal instructions which were easily forgotten.

Many caregivers however pointed out that the Tibamal packaging instructions were difficult to understand for those who were illiterate and stated that it was also important for the retailer to give instructions.

#### Affordable pricing

When asked about affordability, the majority of both retailers and caregivers stated the price was *“not a scary one”* but *“very good”*, *“cheap”* and affordable by most in the community, catering even to those whose *“source of income is very low”*. Almost all retailers expressed satisfaction with the price and stated that it was the *“cheapest antimalarial”* from which they made *“more than 100% profit”*. They added that the community was being helped a lot because one could *“sell two or three eggs and buy the drugs for the child”*. Caregivers added that one could easily borrow Ksh. 20 from a friend to buy the drugs and then pay back.

*“…they are cheap because you see even mothers may be defeated to buy those other drugs or be defeated because the hospital is far, but once she has Ksh. 20 she is sure she can get treatment for malaria for her child.” Retailer, Butere-Mumias.*

However, one caregiver pointed out that, as much as it was affordable, because of varied financial circumstances, a few people may find the price high.

The majority of retailers were happy to have the price printed on the Tibamal packaging as they felt it stopped customers from bargaining and also *“tamed greedy shopkeepers”* who might be tempted to sell above the recommended price.

*“You see, in a shop.....you find if something is in high demand, someone can take that chance to increase the price. But if the price is known someone cannot increase it. Because if your child is sick and I tell you this is the drug, it's Ksh. 100, what will you do? Will you not buy? But now this one has helped so much. The customer will tell you “is it not 20 shillings?”” Retailer, Teso.*

A couple of retailers said they had heard of cases where shopkeepers had taken off the outer packaging on which the price was printed, and sold the drugs at Ksh. 100–120, or even taken the tablets out of the packaging altogether, selling them loose, but this was believed to be rare.

Mixed views were expressed on the two age packs having the same price. Those in favour thought it was practical and fair as all children under five were treated for the same price. They were concerned that changing the price of either pack would lead to inappropriate dosing as caregivers tried to save on costs, or retailers tried to increase sales of the higher priced pack. Those against the uniform price felt that the price of the smaller pack should be decreased as it contained fewer tablets. It was further noted that some caregivers bought a blue pack stating that they had a child above 3 years in order to get extra tablets, when they actually planned to use the pack for a younger child.

*“When a child gets sick the mother tells you the baby is three years and some months and later you will discover that this baby is one between 3 months to 3 years. So she buys this because she wants to use 6 tablets and keep 6 tablets… So a person sees she has used Ksh. 10 for one dose and Ksh. 10 for another.” Retailer, Teso.*

In sum, the intervention was very positively perceived by both retailers and caregivers. Respondents praised the focused retailer training, affordable pricing, strong promotional activities, drugs, dispensing job aids used by retailers, and consumer friendly packaging. This provides explanations for the positive results seen in terms of access, coverage and appropriate pricing. However, despite the positive views on the intervention, for several key outcomes of the intervention there was substantial room for improvement: a high proportion of outlets did not stock Tibamal, advice to consumers was poor, referral was very rare, adherence was sub-optimal, diagnostic use was limited and coverage could have been further increased. The next section explores potential reasons for these limitations.

### Potential explanations for intervention limitations

#### Not all trained staff stocked Tibamal

As noted above, data from retail surveys showed that about a third of trained retailers were not stocking Tibamal [19]. The most common reasons given for this by retailers were that some businesses had closed due to lack of capital and in others the person trained had left the business, leading PSI to stop supplying this outlet. Other businesses were said to have relocated or ceased selling drugs.

Another factor was the unreliability of drug supplies from PSI which was the source of much dissatisfaction among retailers and caregivers. The majority felt that supplies were insufficient, describing them as *“scarce”* or *“in short supply”*, a situation they felt was worsened when health facilities experienced stock-outs. Retailers complained that deliveries were infrequent, at times more than a month apart, hence not keeping pace with increasing demand as the promotion took effect. Communication about delivery timings was said to be poor, and no credit was allowed which was problematic as retailers did not always have sufficient cash to hand. In addition, PSI placed restrictions on the quantity one could purchase at a time.

*“Sometimes they only give two packets even when you have money you are told everyone is to get two, two. That is two for small children and two for older children aged 3–5 years… it is always a problem after a short while. Because you can sell that in two days and it's out of stock and the drugs are brought at the end of the month….as we speak, I don't think anyone has Tibamal now.” Retailer, Teso.*

Finally, as noted above, in a few instances PSI was said to have stopped supplying a few retailers who failed to record all the required details in the DAR.

#### Caregiver counselling sub-optimal

The mystery shopper survey demonstrated that retailers dispensing Tibamal were poor at giving their clients appropriate advice [20]. FGD results were consistent with this, with almost all caregivers stating that they did not get much advice when the drugs were dispensed. Advice given was mainly only on how to give the drugs, with advice on care of the child if they vomited very rare.

Retailers admitted that advice was given less frequently compared with the outset of the programme. A major reason mentioned was time. The majority of retailers, especially those from general stores, stated that in many instances, they would *“have many customers to attend to”* and hence not have *“time to discuss with a patient to satisfaction”*. Many others blamed their customers, stating that they would come to the outlets in a hurry and become impatient, refusing to wait for instructions, or would send young children to the outlets who could not understand the advice. Other reasons given were that some retailers were poorly educated or had low literacy levels and would thus forget what they were taught due to lack of refresher courses.

Many caregivers felt that advice given was worse in general stores as compared to drug shops because in general shops *“their work is to sell”* and that a shopkeeper may be confused unlike in drugs stores where they were trained. In reference to general stores, one caregiver from Busia stated, *“They only ask you, “is your child sick? Eee…what drug do you want?”…He gives you and asks you to go home.”* One retailer was of the strong opinion that general shopkeepers should not be allowed to sell the drugs as they would not do so in an appropriate manner.

*“I think if you give Tibamal to a shopkeeper, how do you expect to get good results? …I think it was not practical. I think they should take people with medical experience.” Retailer, Butere-Mumias.*

On the other hand, those from areas with none or few specialized drug stores disagreed, stating that as long as one had been trained and mandated to sell the drugs there was no problem.

Moreover, mystery shopper findings demonstrated that the difference in performance by shop type was not clear-cut. Specialized drug stores were more likely than general shops to give appropriate advice on how to administer the drugs (46% v. 24%) but general stores were somewhat more likely to give appropriate advice on what to do if the child did not get better (37% v. 27%). Advice on what to do if the child vomited and the appropriate foods to give was extremely rare in both outlet types [20].

#### Tibamal dosing adherence sub-optimal

The household survey demonstrated that adherence by patients to AL dosing guidelines was inadequate at about 70%, though similar to adherence to AL from health facilities [21]. During FGDs a fairly large number of caregivers admitted to not always adhering to dosing guidelines. A major reason given was that the child responded very fast to Tibamal and started playing again which led them to stop the drugs and *“save them for another day”*. Another reason was that caregivers would share one pack of drugs between two children either if the second got sick at night or if they lacked money to buy more drugs.

*“… even me I do that… I can give the child Tibamal immediately they catch a fever, and on the second day you see the child has started playing… then I put away the remaining tablets and wait for another day when they get sick, that is the one I begin with before I go to buy another one.”Caregiver, Teso.*

The time interval of 8 hours between the first and second doses was also highlighted as a reason for poor adherence, especially when the second dose was expected to be given in the middle of the night. Most retailers were very clear on the dosing guidelines for each of the Tibamal packs but quite a few caregivers on the other hand, were not. Discrepancies were especially seen in the correct ages for each of the packs, with some mentioning ages of one year to 3 years or from one month to 3 years for the yellow pack and 4 to 5 years for the blue, or even up to 6 years.

One caregiver felt that a lot of caregivers had become *“lazy”* when it came to giving drugs to their children. Many retailers concurred and stated that caregivers complained to them that the drugs were too many and the dose was too long and hence caregivers would get tired and view giving the drugs as a *“burden”*.

*“…these people complain… those drugs that they are too many. Most of them see that they need a drug that they give a child once or take it once….not that they swallow in the morning, evening and other times. To them it seems like a burden or too much work.” Retailer, Butere-Mumias.*

Another reason raised by a minority of caregivers was that some children had difficulty swallowing tablets and would spit them out or vomit. When this occurred several times, some would *“give up”* and stop giving the drug. A few others added that the drugs had a bitter taste that the children did not like. It was thus suggested that the drugs be prepared in different forms other than tablets, such as syrups, injections or suspensions, to make them easier to give to younger children.

*“…honestly I stopped…I cannot speak lies….I went and gave my child the drugs, when I gave he vomited…I gave again he vomited…I gave again he vomited…so I gave up and decided to just stop…” Caregiver, Busia.*

#### Rare referrals to health facilities by retailers

During the training sessions, retailers were taught to refer any child with signs of severe disease or who did not recover after initial treatment with Tibamal, using CHW referral forms. A large majority reported never having used the form as most customers did not come back to the outlets if their children did not get well, an observation confirmed by some caregivers. One retailer from Teso said of the form *“I have never even touched it”*. The retailers pointed out that another reason for low use of the referral form was because the *“drug is effective”*.

#### Tibamal diversion to older age groups

Household survey data showed that Tibamal was obtained in only 6.7% of fevers in those aged 5 years and above in the intervention arm, indicating relatively low levels of diversion to non-target age groups [23]. However, during FGDs it was commonly reported that some customers acquired Tibamal for older patients. They used various methods, such as buying from two outlets, claiming to have twins or two sick children, or sending different people at different times to the same or different outlets.

*“… I just went and bought the drugs, two blue ones and I gave to a child of ten years…when I went back to buy the second, he asked me why I was using another packet, so I asked them “how many children do I have? I have two children, just give me the drug” …. I wanted to reach the right dose in my mind, one packet would not have been enough, so I used two packets and the child got well and went back to school…”Caregiver Teso.*

Reasons given by both retailers and caregivers for such diversion were the periodic AL stock outs at government facilities, and the high cost of other AL drugs available for adults in private retailers. There were unanimous calls for Tibamal to be supplied in pack sizes for older patients.

#### Low utilisation of diagnostics

Utilisation of malaria diagnostics was very low at about 8% of childhood fevers [23], reflecting low use of microscopy and RDTs at public health facilities [10], and the lack of a diagnostic component in the Tibamal intervention, which was based on presumptive treatment. Most retailers and caregivers expressed awareness and some use of blood slides for malaria testing at health facilities, but only two caregivers reported having been tested using an RDT. Low use did not reflect an unwillingness to use the technology, with almost unanimous agreement that RDTs should be supplied in public and private outlets, as having the tests would ensure treatment was provided after a confirmed diagnosis.

*“It will be something to confirm that the child has malaria. You won't just rely on the signs and symptoms, you will make sure.”Retailer, Busia.*

However a key concern raised was affordability. The majority of caregivers felt that the tests should be *“at an affordable price….maybe even 20 shillings* (0.25 USD)*”*.

When shown samples of RDTs, another concern raised was that a large majority of retailers and caregivers thought they were similar to rapid HIV tests used at health facilities. Many felt that some people would fear the test was for HIV, and therefore decline it, pointing out the need for rigorous community awareness before the introduction of RDTs. Concerns were also raised on hygiene, quality assurance and approved stockists. A large majority of caregivers felt that RDTs should only be made available at drug stores and health facilities. They felt strongly that in general stores the retailers would not be able to sell other goods to people and still have time to explain and carry out the tests.

## Discussion

Findings from the FGDs helped reinforce and explain key results from the quantitative household, provider and mystery shopper surveys, providing insight into the factors that both led to and limited the success of the intervention. Key to the success of the intervention was the focused shopkeeper training which helped prepare the retailers to be adept and confident in dispensing the drugs. The drugs were also sold at an affordable price to both the retailers and consumers which was vital in an area where more than half the population live below the poverty line. Retailers selling Tibamal reported realizing good profits from the sale of the drugs and improved sales in many items in their outlets, incentivising their participation. The promotional activities in the community ensured that awareness was created and drug price clarified, leading to high demand for Tibamal, and good adherence to the recommended retail price. Consumer friendly information on Tibamal packaging supplemented the advice given by retailers, and job aids helped ensure that most clients received the correct dose. This was reflected in the household survey, where at follow up in the intervention group over 80% of those receiving Tibamal received the correct dose and over 95% paid the recommended retail price [21]. Similar positive effects on ACT use have been seen in other ACT subsidy programmes [30]. Prior to the introduction of ACTs, previous private sector interventions employing similar strategies, such as focused training, use of job aids and provision of consumer information, have also been effective in improving drug selling practices and consumer knowledge and behaviour [29], [31], [32].

Though the intervention led to significantly improved ACT coverage, its success was limited by a number of factors. Although trained, many retailers were not stocking Tibamal, due to lack of capital, staff turnover and change in or relocation of the business. An additional important constraint was lack of consistent and sufficient Tibamal supplies. This corroborates findings from the provider survey that showed that 32.5% of Tibamal trained outlets reported stock outs of AL within the previous 2 weeks [19]. In the mystery shopper survey at follow-up sales of Tibamal were reported in 58% of outlets which had stocked Tibamal during the provider survey, compared to only 25% in all outlets in the intervention arm [20]. This demonstrates that with regular and more consistent supplies, a potentially higher increase in ACT coverage could have been realized.

Retailers performed poorly on providing advice to patients, explaining that both they and their clients sometimes had insufficient time for this, and that some instructions had been forgotten. Adherence by caregivers to dosing guidelines was also sub-optimal, because of a wish to save tablets for other children or illness episodes, doses being required at night, a tendency to stop giving the tablets immediately the child felt better, and the number and bitter taste of the tablets. Similar reasons for non-adherence have been identified in other settings [12], [33]. The bitter taste could have been countered if the cherry tasting water-dispersible AL drug for children had been available at the time of the study. Finally the DAR forms and referral forms appeared to have little impact on quality of care and the intervention design could have been simplified by their removal.

The FGDs also served to highlight a behaviour that may have been under-reported in the quantitative data. In the household survey, Tibamal was reportedly obtained in only 6.7% of fevers in those 5 years and above in the intervention arm [23]. However, in FGDs the diversion of Tibamal to these groups was frequently raised, indicating that it may have been more common. It is possible that participants felt more confident discussing such diversion in the FGDs, as they did not have to reveal anything about their own behaviour. On the other hand it may be that this issue was frequently discussed in FGDs simply because it was perceived as an interesting topic.

Several limitations in the FGD methods should be noted. Firstly, due to financial and time constraints the retailer FGDs in each sub-location included both men and women, and staff from both general and specialized drug stores. It might have been preferable to have interviewed the genders and outlet types separately so that they felt less inhibited in their responses. This would also have allowed us to distinguish between general store and specialized drug store respondents in the analysis. Secondly, the participants were told to feel free to use their mother tongue or whichever language they felt comfortable in, but the interviews were mainly carried out in Swahili. Although most respondents use Swahili on a daily basis, it is possible that a few found it difficult to express themselves fluently. Finally, having been recipients and beneficiaries of the intervention, there may have been a tendency for respondents to present the intervention in a positive light to ensure its continuation. Notably though, as much as some may have felt uncomfortable talking about inappropriate behaviour, many were open and ready to cite negative aspects of the intervention.

The study took place in only three districts in Western Kenya, and the intervention design was constrained due to the cluster randomised design, which meant that mass media could not be used, and subsidised drugs had to be delivered directly to outlets rather than going through the normal distribution chain. However, the findings highlight a number of factors that can be cautiously applicable to other settings and subsidy programmes, such as the AMFm. Implementation of the latter began in seven countries in 2010/11, and involves an ACT subsidy at the top of the global supply chain for products distributed through the public, private for-profit and private not-for-profit sectors [18]. In Kenya subsidised AL is being distributed at a maximum retail price of 0.49 USD (Ksh. 40), supported by training and a mass media campaign. Officially the only retailers allowed to stock AMFm ACTs are registered pharmacies, although they are often sold through unregistered outlets [34]. Results from a recent evaluation found that implementation of AMFm in Kenya was associated with a 34 percentage point increase in availability of quality-assured ACTs (QAACTs) in outlets stocking antimalarials, and a 31 percentage point increase in QAACT market share overall, and a 49 percentage point increase in market share in the private for-profit sector [35].

The findings from the FGDs have a number of implications for such subsidy programmes:

- Price – A price of 0.25 USD (Ksh. 20) for a child dose was generally affordable in these very poor communities, indicating that if the maximum AMFm price of 0.49 USD (Ksh. 40) for an adult dose is adhered to, demand is likely to be high.
- Community mobilisation – Awareness raising activities for the community are popular and important, but must include considerable emphasis on patient adherence to the full treatment dose.
- Outlet type – Although general and drug stores performed equally well on key quantitative indicators, some caregivers favoured the restriction of subsidised products to specialised drug shops, and it was generally agreed that only drug stores should be allowed to provide RDTs.
- Retailer training & job aids – Simple posters explaining dosage regimens are popular among retailers and felt to improve dispensing. Retailers greatly appreciated a short training course and felt that it helped them gain vital skills and confidence. Although previous experience has demonstrated the feasibility of delivering large scale retailer training programmes in similar settings [36], this is constrained under AMFm as only registered pharmacies are officially allowed to dispense this prescription only medicine in Kenya. This is likely to further compromise correct dispensing and advice from many specialised drug stores, as registered pharmacies are very limited in number and mainly restricted to urban areas.
- Counselling of caregivers – the provision of accurate advice to caregivers on case management is a major challenge. This could be addressed to some degree through more frequent refresher training, but perceived time constraints mean this is likely to remain suboptimal. An alternative would be to provide these key messages to caregivers through the community mobilisation campaign.
- Diversion to non-target groups – if subsidised drugs are provided to certain age groups only, there is likely to be diversion to non-target groups, and this may be underestimated in quantitative surveys. Under AMFm subsidised drugs are provided for all age groups, so such diversion will not take place, although this raises the concern that a high proportion of the subsidy is spent on age groups at less risk of severe disease and death [37].
- Diagnosis – It is now Kenyan policy that a confirmed diagnosis be made prior to antimalarial treatment [38], though implementation is far from complete even in the public sector. This study showed that retailers and caregivers would eagerly accept RDT provision through drug stores as long as the test was clearly for malaria, it was cheap, and proper training and quality controls were in place.

## Conclusions

This study has highlighted that important components of a successful ACT subsidy intervention are regular retailer training, affordable pricing, a reliable supply chain and community mobilization emphasizing patient adherence and when to seek further care. Further research is needed on caregiver and retailer perceptions of larger scale subsidy programmes such as the AMFm, and on strategies to improve counselling by providers, adherence by patients and the expansion of diagnostic coverage in the retail sector.
